# Two-stage steam explosion pretreatment of softwood with 2-naphthol as carbocation scavenger

**DOI:** 10.1186/s13068-019-1373-3

**Published:** 2019-02-21

**Authors:** Christoph-Maximilian Seidel, Simone Brethauer, László Gyenge, Philipp Rudolf von Rohr, Michael H. Studer

**Affiliations:** 1 Institute of Process Engineering, ETH Zürich, Sonneggstrasse 3, 8092 Zurich, Switzerland; 2 School of Agricultural, Forest and Food Science, Bern University of Applied Science, Länggasse 85, 3052 Zollikofen, Switzerland; 3 Present Address: Faculty of Economics and Socio-Human Sciences and Engineering, Sapientia - Hungarian University of Transylvania, Miercurea Ciuc, Piaţa Libertăţii nr. 1, 530104 Cluj-Napoca, Romania

**Keywords:** Spruce, Bioethanol, Simultaneous saccharification and fermentation, Enzymatic hydrolysis, Steam explosion pretreatment, Carbocation scavenger, 2-Naphthol, Inhibition

## Abstract

**Background:**

Lignocellulosic biomass is considered as a potential source for sustainable biofuels. In the conversion process, a pretreatment step is necessary in order to overcome the biomass recalcitrance and allow for sufficient fermentable sugar yields in enzymatic hydrolysis. Steam explosion is a well known pretreatment method working without additional chemicals and allowing for efficient particle size reduction. However, it is not effective for the pretreatment of softwood and the harsh conditions necessary to achieve a highly digestible cellulose fraction lead to the partial degradation of the hemicellulosic sugars. Previous studies showed that the autohydrolysis pretreatreatment of softwood can benefit from the addition of 2-naphthol. This carbocation scavenger prevents lignin repolymerisation leading to an enhanced glucose yield in the subsequent enzymatic hydrolysis.

**Results:**

In order to prevent the degradation of the hemicellulose, we investigated in this study a two-stage 2-naphthol steam explosion pretreatment. In the first stage, spruce wood is pretreated at a severity which is optimal for the autocatalytic hydrolysis of the hemicellulose. The hydrolyzate containing the solubilized sugars is withdrawn from the reactor and the remaining solids are pretreated with different amounts of 2-naphthol in a second stage at a severity that allows for high glucose yields in enzymatic hydrolysis. The pretreated spruce was subjected to enzymatic hydrolysis and to simultaneous saccharification and fermentation (SSF). In the first stage, the maximal yield of hemicellulosic sugars was 47.5% at a pretreatment severity of log $R_0$ = 3.75 at 180 °C. In the second stage, a 2-naphthol dosage of 0.205 mol/mol lignin C_9_-unit increased the ethanol yield in SSF with a cellulose loading of 1% using the whole second stage pretreatment slurry by 17% from 73.6% for the control without 2-naphthol to 90.4%. At a higher solid loading corresponding to 5% w/w cellulose, the yields decreased due to higher concentrations of residual 2-naphthol in the biomass and the pretreatment liquor, but also due to higher concentrations of potential inhibitors like HMF, furfural and acetic acid. Experiments with washed solids, vacuum filtered solids and the whole slurry showed that residual 2-naphthol can inhibit the fermentation as a single inhibitor but also synergistically together with HMF, furfural and acetic acid.

**Conclusions:**

This work shows that a two-stage pretreatment greatly enhances the recovery of hemicellulosic sugars from spruce wood. The presence of 2-naphthol in the second pretreatment stage can enhance the ethanol yield in SSF of steam explosion pretreated softwood at low cellulose concentrations of 1% w/w. However, with higher solid loadings of 5% w/w cellulose, the ethanol yields were in general lower due to the solid effect and a synergistic inhibition of HMF, furfural, acetic acid with residual 2-naphthol. The concentration of residual 2-naphthol tolerated by the yeast decreased with increasing concentrations of HMF, furfural, and acetic acid.

**Electronic supplementary material:**

The online version of this article (10.1186/s13068-019-1373-3) contains supplementary material, which is available to authorized users.

## Background

In the past years, biofuels as sustainable energy carriers have received increasing interest due to environmental concerns and uncertain oil supply [1–3]. In 2012, the transportation sector accounted for around 25% of the worldwide energy use, whereof 96% (1.06 × 10$^{20}$ J) was supplied by liquid fuels. Despite the increasing electrification of the individual transport, it is projected that liquid fuels will continue to be the largest energy carrier until at least 2040 [4]. Lignocellulosic biomass like wood, energy crops, or agricultural waste is the most abundant raw material on Earth which can be used for the production of biofuels [5].

Softwood is the dominant source of lignocellulose in the Northern hemisphere [6]. The main components of wood are cellulose, hemicellulose, and lignin. The cellulose is located inside the cell walls and is naturally protected by a complex matrix of lignin and hemicellulose. The hemicellulosic fraction of softwood is mostly built from mannose (a hexose), which can be fermented together with glucose derived from cellulose by enzymatic hydrolysis by e.g., bakers yeast [6] to ethanol or by a different microorganism to the desired chemical. Lignin is usually burned for steam and power production, but might also be used for the production of aromatic chemicals [7].

The biochemical conversion process of lignocellulose to biofuel starts with a pretreatment step that is necessary to obtain high yields of fermentable sugars in the enzymatic hydrolysis step [6, 8]. The pretreatment reduces the biomass’ recalcitrance by chemical and/or physical changes in the biomass structure.

Hydrothermal or autohydrolysis pretreatments are in general very attractive compared to other methods [9] as no additional chemicals except water are required, which makes neutralization or removal of organic solvents obsolete, resulting in a simplified downstream process [10]. Steam explosion (SE) pretreatment is one type of autohydrolysis pretreatment that was tested on a commercial scale [11]. The technology allows for high biomass loadings in the pretreatment reactor [10, 11] and can also deal with large particle sizes [12–14].

For softwood, however, hot water pre-treatment (HWP) or SE as well as ammonia fiber expansion (AFEX) are not very effective [15, 16]. It has been shown that the use of an acid catalyst in HWP or SE can be applied to overcome the recalcitrance of softwood. One possibility is to impregnate softwood with sulfur dioxide, which is absorbed by the moisture in the biomass to form sulfurous acid prior to the steam explosion [6, 17, 18]. Another pretreatment technology, which also uses an acidic catalyst, is the sulfite pretreatment to overcome recalcitrance (SPORL). The biomass is pretreated with a sulfite solution at medium temperatures (160–180 °C) and then milled to obtain a fibrous material [15]. Both pretreatment methods lead to high sugar yields in the subsequent enzymatic hydrolysis, but the use of an acid catalyst causes high equipment corrosion and a high formation of toxic fermentation inhibitors [15, 19]. If gaseous SO_2_ is used, its toxicity is also a concern [19].

Alternatively, the high recalcitrance of softwood can also be overcome by adding 2-naphthol to the aqueous pretreatment. 2-Naphthol acts as a carbocation scavenger that suppresses lignin repolymerisation [20], which leads to less condensed lignin structures [21]. Celluloytic enzymes adsorb non-productively on condensed lignin structures [22] and by the prevention of lignin repolymerisation the enzymatic cellulose digestibility can be improved. For example, by the addition of 0.205 mol 2-naphthol per mol lignin C_9_ unit, the enzymatic cellulose hydrolyzability can be increased by more than 50% to quantitative conversion after a one-stage steam explosion pretreatment at log $R_0$ = 5 [23].

One disadvantage of steam explosion pretreatment is that the hemicellulose fraction is partially degraded at high pretreatment severities [10] that are required to enhance the cellose digestibility [24]. The degradation of hemicellulosic sugars results in a reduced yield of fermentable sugars but also leads to the formation of enzyme and fermentation inhibitors such as 5-hydroxymethylfurfural (HMF), furfural and acetic acid [25]. To maximize the yield of both hemicellulosic and cellulosic sugars and thus maximize the total ethanol yield, a two-stage pretreatment can be applied [26]. In this process, the hemicellulose is solubilized at a moderate severity in a first pretreatment step and withdrawn from the reactor. The remaining solids are then pretreated in a second stage at a higher severity, which allows for a high glucose yield in the subsequent enzymatic hydrolysis.

In this work, a two-stage steam pretreatment process was investigated, where in the first stage the yield of soluble sugars from the hemicellulose fraction was maximized and in the second stage 2-naphthol was added in order to increase the enzymatic cellulose digestibility. We also studied the influence of different 2-naphthol concentrations in the pretreatment on subsequent simultaneous saccharification and fermentation (SSF) for the production of ethanol from softwood. This is of interest as the addition of 2-naphthol at concentrations higher than 0.1 g/l led to reduced ethanol yields in glucose fermentations [27]. To elucidate the effect of 2-naphthol added to the pretreatment stage, SSF experiments were performed at a cellulose concentration of 1 and 5% w/w using washed solids, filtered solids, and whole pretreatment slurries.

## Material and methods

### Biomass

Spruce was cut in August 2016 in Lohn-Ammansegg (canton of Solothurn, Switzerland) from a roughly 50 year old tree and chopped with a wood chipper through a 30 mm sieve. After drying at 40 °C in a convection oven, the spruce had a dry matter content of 92.1 ± 0.0% and was milled through a 4 mm sieve (Retsch cutting mill SM100). The composition was: glucan 40.5 ± 0.3%, mannan (including xylan and galactan) $19.3\pm 0.2$%, acid-soluble lignin (ASL) $8.6\pm 0.1$%, acid-insoluble lignin (AIL) $22.2\pm 0.4$%, acetyl $1.5\pm 0.1$%, ash $0.2\pm 0.1$% and water extractives $4.8\pm 0.5$% (total $99.0\pm 1.4$%).

### Steam pretreatment reactor

The pretreatment experiments were carried out in a steam gun set up by the Industrieanlagen Planungsgesellschaft (IAP, Graz, Austria). The reactor is made of stainless steel (type 1.4475) with a volume of 5.8  l. A process scheme and a detailed description can be found elsewhere [28]. For this work an additional filtration device (filter pore size 1 mm, CAD drawing in Additional file 1: Figure S1) was installed in the steam gun to release hydrolysate from the pressurized reactor. Furthermore, an additional liquid feeding device was installed.

### First stage pretreatment

The pretreatment severity parameter $R_0$ was used to monitor and compare the pretreatment severity of the pretreatment experiments [29].

1 $$\begin{aligned} R_0=t/\text {min} \cdot \text {exp}\left[ {\frac{T/^\circ \text {C}-100}{14.75}}\right] \end{aligned}$$

The reactor was first pre-heated for about 1 h at the desired temperature until a stable outside wall temperature was reached. Prior to each experiment the reactor was heated for 10 min at the desired pretreatment temperature. The steam and the condensate were released through the filtration device and the reactor was immediately charged with 400 g biomass (prepared as described below). The countdown of the pretreatment time was started with a tolerance of 1% to the desired pretreatment temperature, which was reached after 100–120 s. After the desired pretreatment time of the first stage, the hydrolysate was released from the pressurized reactor through the filtration device. Then, 1.2 l of water (room temperature) was injected (under reaction pressure) and immediately released through the filtration device in order to wash the solids. The pressure was reduced to 3 barg through an exhaust valve in the upper part of the reactor followed by the release of the biomass through a ball valve at the bottom without an explosive decompression. In order to find the optimal conditions for hemicellulose recovery, experiments were performed at seven different pretreatment severities between 3.25 and 4.5 at four different pretreatment temperatures from 170 to 200 $^\circ$C (Table 1). During the optimization procedure, only the liquid phase was analyzed and the solids were discarded. After choosing an optimal condition for stage 1, only biomass which was pretreated under this condition was used for the second stage pretreatment experiments. For each experiment in the second stage, one pretreatment run was performed in stage 1 as described above. The resulting pretreatment slurry was vacuum filtered through Whatman No. 1 filter paper and the solids from all experiments were pooled to be further processed in the second stage experiments. The pretreated cellulose enriched solids were not further dried or washed prior to further proceeding with the impregnation with 2-naphthol.

**Table 1:** Overview of experiments for the optimization of the first pretreatment stage

Temp (°C) (p [barg])	Pretreatment severity log $R_0$ [−]						
	3.25	3.5	3.75	4	4.25	4.5	4.75
170 (7)	x	x	x				
180 (9)		x	x	x			
190 (11.5)			x	x	x	x	
200 (14.5)				x	x	x	x

Indicated with an x is at which severity and temperature an experiment was performed. Corresponding vapor pressure displayed in brackets

### Biomass impregnation with 2-naphthol

Pretreated biomass from stage 1 was impregnated with different amounts of 2-naphthol dissolved in ethanol. The amount of 2-naphthol was calculated based on the lignin content of the raw biomass and dosages of 0.205, 0.1025 and 0.05125 mol 2-naphthol per mol lignin C_9_-unit were chosen (with an assumed molecular weight of a lignin C_9_-unit of 184 g/mol [30]). The desired amount of 2-naphthol was dissolved in 2 l of ethanol and the biomass was soaked in this solution for 48 h. During this procedure the biomass solvent mixture was mixed by hand regularly and it was placed into a fume hood to evaporate the ethanol at the same time. After the evaporation of the ethanol supernatant, the samples were not further dried. A control sample without ethanol or 2-naphthol was placed besides the impregnated samples into the fume hood.

### Second stage pretreatment

The preheating of the steam pretreatment reactor was done analogously to the first stage pretreatment. First stage pretreated and 2-naphthol impregnated biomass as well as the control sample was pretreated at a severity of log $R_0$ = 5 (*T*= 230 °C, *t* =  5 min, *p* = 27 barg), which has been shown in a former work to be optimal for enzymatic cellulose digestibility [23]. After the desired pretreatment time, the biomass was rapidly discharged. The obtained biomass slurry was weighed and vacuum filtered through Whatman No. 1 filter paper, and the volume, weight, and pH of the filtrate were recorded. A part of the filter cake was extensively washed with water (20 times the weight of the biomass at room temperature). All pretreatment experiments were performed in duplicates and both samples were mixed thoroughly.

### Enzymatic hydrolysis

The enzymatic hydrolysis of the biomass was conducted in 20 ml scintillation vials with a sample size of 10 ml according to the NREL standard procedure using a cellulose concentration of 1% w/w [31]. The following changes were made: Sodium azide with a final concentration of 0.01 g/l was used instead of antibiotics and the pH of the 0.05 M sodium citrate buffer was adjusted to 5.0. Accelerase 1500 (DuPont; batch number 4902279303) with an activity of 45 filter paper units (FPU) per ml as determined according to the NREL standard procedure [32] was used with different enzyme loadings between 5 and 60 FPU/g cellulose. The samples were incubated in a shaker (Minitron; Infors-HT) with a shaking throw of 50 mm at 50 °C and 200 rpm for 120 h and the sugar concentrations were analyzed in the supernatant. All hydrolysis experiments were carried out in triplicate and single standard deviations are reported with the mean.

### Simultaneous saccharification and fermentation (SSF)

**Fig. 1:**
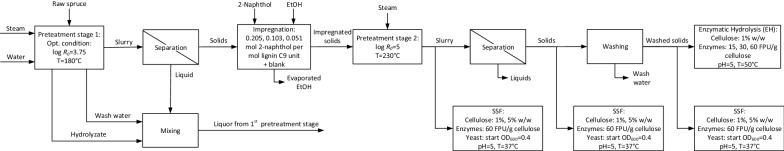
Basic flow sheet of the experimental procedure

SSF experiments were conducted according to the NREL standard procedure [33] using a final cellulose concentration of 1% w/w and 5% w/w in a citrate buffered growth medium containing 20 g/l peptone and 10 g/l yeast extract employing 100 ml crimp neck vials equipped with a needle for the escape of CO$_2$ and the withdrawal of samples. Sterile filtered Accelerase 1500 (DuPont; batch number 4902279303) with an activity of 45 filter paper units (FPU) per ml was used at a final concentration of 60 FPU/g cellulose. Distillers yeast (VTT C-79092) was grown from cryostocks on a medium containing 20 g/l peptone, 10 g/l yeast extract and 3 g/l maltose. The SSF samples were inoculated with a final OD_600_ of 0.4. The samples were incubated in a shaker (Multitron; Infors-HT) with a shaking throw of 25 mm at 37 °C and 130 rpm for 10 days. During this period, samples were withdrawn periodically and analyzed for sugars and ethanol in the supernatant. Three different substrates types were used: washed pretreated solids, vacuum filtered solids, and the whole second stage pretreatment slurry (Fig. 1).

### Analytical methods

The analysis of the dry matter content, the biomass composition and the content of mono- and oligomeric sugars in the pretreatment liquor and of monomeric sugars in the enzymatic hydrolysis liquor were done according to the laboratory analysis methods from the National Renewable Energy Laboratory (NREL) [34–38]. A Waters 2695 separation module equipped with a Bio-Rad Aminex HPX-87H column with pre-column and a Waters 410 differential refractometer were used for the analysis of sugars, ethanol, and inhibitors. The column was operated at 60 °C with a flow of 0.6 ml/min 0.005 M sulfuric acid. All biomass and pretreatment liquor analyses were performed in triplicate and duplicate, respectively. Mean values with single standard deviations are reported in this work.

### Quantification of unreacted 2-naphthol

To determine the amount of unreacted 2-naphthol, the 2-naphthol remaining in the pretreatment liquor was extracted and analyzed by gas chromatography/mass spectrometry (GC/MS). 5 ml water was added to 10 ml pretreatment liquor and the mixture was extracted three times with 3 ml CHCl$_3$. To determine remaining 2-naphthol in the biomass, 5 ml water was added to 10 g washed or filtered biomass and extracted three times with 10 ml CHCl$_3$.

Two hundred and fifty microlitre of a syringaldehyde solution as internal standard was mixed with 750 μl of 2-naphthol extract. The concentration of the syringaldehyde solution was 0.5 g/l or 1 g/L for pretreament liquor extracts and biomass extracts, respectively. An autosampler (Thermo Scientific, AI 3000, Waltham, MA, USA) was used to inject the samples (5 μl for the extracts of the liquor and 1 μl for the extract from the biomass) into the GC/MS system (Thermo Scientific, Trace GC Ultra/Polaris ITQ ion trap, EI mode). The split ratio for the injections was 10:1. The GC system was equipped with a Supelco SLB 5 ms capillary column (30 m × 0.25 mm × 0.25 μm). Helium was used as the carrier gas with a flow of 1 ml/min. The following temperature program was used for the GC oven: 80 °C for 5 min, heating by 10 K/min to 280 °C, and 280 °C kept for 5 min.

## Results and discussion

### Optimization of first stage pretreatment

To maximize the recovery of hemicellulosic sugars in the first stage pretreatment and minimize the formation of inhibitors, pretreatment experiments at four different temperatures were performed at seven different pretreatment severities (see Table 1).

**Fig. 2:**
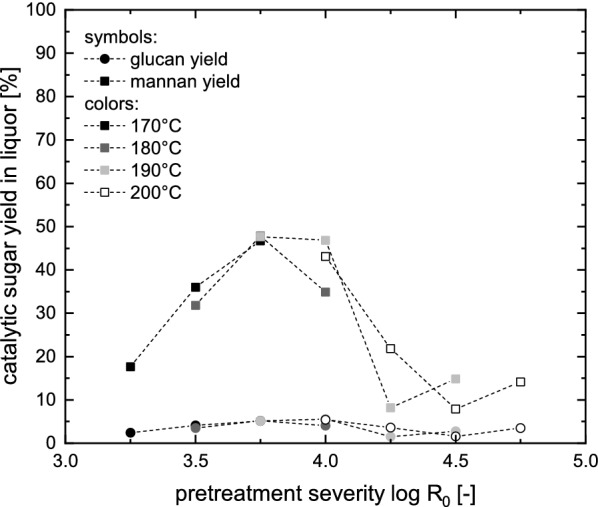
Recovery of sugars from cellulose and hemicellulose in the first stage pretreatment hydrolysates

The combined pretreatment hydrolysate including the washing water was analyzed for dissolved sugars from cellulose and hemicellulose. The resulting glucan and mannan recoveries (sum of mono- and oligomeric sugars) in the pretreatment liquor are presented in Fig. 2.

The maximum mannan yield was 47.5% reached at a pretreatment severity of log $R_0$ = 3.75 at temperatures of 180 and 190 °C. At this severity, the glucan yield was with 5% identical for both temperatures. Further information about the proportion of monomeric sugars can be found in Additional file 2: Figure S2. Typical mannan yields in the pretreatment hydrolysate of one-stage pretreatments that aim at maximizing the enzymatic cellulose conversion are between 5 and 10 % [28]. In studies were an acidic catalyst (sulfuric acid or sulfur dioxide) was used in two-stage steam explosion pretreatments, 60–80% of the hemicellulose fraction can be recovered [18, 39]. However, both of these studies included an extensive washing step between the two pretreatment stages, therefore the hemicellulosic sugars are partly obtained in very low concentrations in the wash water. We selected a pretreatment temperature of 180 °C and a severity of $R_0$ = 3.75 for the first stage of the two-stage pretreatment of spruce. At this condition, multiple pretreatment batches were run in order to obtain enough biomass for investigating and optimizing the second pretreatment stage. After pooling the pretreatment batches, they had a dry matter content of $21.3\pm 1.2$%.

### Enzymatic hydrolysis

**Fig. 3:**
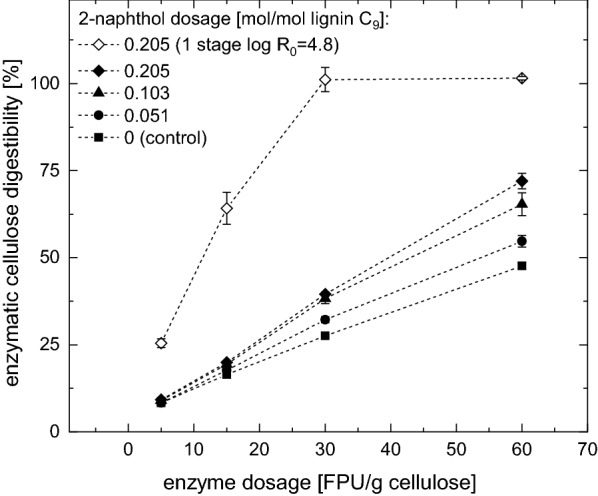
Influence of different amounts of 2-naphthol on the enzymatic cellulose digestibility of washed pretreated spruce wood at different enzyme loadings. Pretreatment conditions: first stage: *T *= 180 °C, log $R_0$ = 3.75; Second stage: *T * = 230 °C, log $R_0$ = 5. Hydrolysis conditions: t = 120h, *T * = 50 °C, 1% w/w cellulose. One-stage pretreatment as comparison: *T *= 230 °C, log $R_0$ = 4.8, 0.205 mol 2-naphthol/mol lignin C_9_-unit

Steam explosion pretreatment experiments with 2-naphthol were up to now only performed in a one-stage pretreatment process [27]. In order to investigate if the enhancement of the cellulose digestibility by adding 2-naphthol can be reproduced with a two-stage pretreatment process, enzymatic hydrolysis experiments were performed with washed pretreated spruce wood solids (Fig. 3). Results are shown for pretreatments using different amounts of 2-naphthol (0, 0.051, 0.103, and 0.205 mol/mol lignin C_9_-unit) and for different enzyme loadings (5, 15, 30, and 60 FPU/g cellulose). For comparison, results of the enzymatic cellulose conversion of spruce wood chips, which were pretreated in a one-stage pretreatment at a severity of log $R_0$ = 4.8 with 0.205 mol 2-naphthol mol lignin C_9_-unit are presented.

The enzymatic cellulose conversion increases with increasing enzyme dosages as well as with increasing amounts of 2-naphthol. At the highest enzyme dosage of 60 FPU/g cellulose, the enzymatic digestibility could be increased from 47.7 ± 0.9% for the 2-stage pretreated control to 72 ± 2.3% for the material pretreated in the presence of 0.205 mol/mol 2-naphthol, corresponding to an improvement of 24.3%. After a one-stage pretreatment at a severity of log $R_0$ = 4.8 of spruce wood chips impregnated with 0.205 mol 2-naphthol per mol lignin C_9_-unit complete enzymatic cellulose conversion was reached already with an enzyme dosage of 30 FPU/g cellulose. This is in good agreement with Pielhop et al. [23], who reported similar digestibilities for spruce wood pretreated in one stage with the same amount of 2-naphthol. One possible reason for the much lower glucose yield after a two-stage pretreatment could be that the partial removal of the hemicellulose during the first stage decreases the amount of catalytically active acetic acid that is released during the pretreatment. However, a pH of $\sim$ 3.2 (see Table 2) was measured in both the one-stage and the two-stage pretreatment hydrolyzates [23] indicating similar acetic acid concentrations. Another hypothesis is that the high water content (78.7%) in the biomass after the first stage pretreatment reduces the pretreatment efficiency in the second stage. With a higher water content inside the biomass it takes longer for the water inside the biomass to reach the reaction temperature, even if the heat-up times are very similar [23]. This phenomenon is currently investigated in more detail in our laboratory. It is reported that in dilute acid steam explosion pretreatment, the sugar yield is increased to a great extend when pretreating in two steps instead of one [39]. However, when the biomass is impregnated with dilute sulfuric acid prior to each pretreatment step, there is no difference in water content, which could explain that.

**Table 2:** Pretreatment liquor composition of the first stage pretreatment and of the second stage with different 2-naphthol dosages

	1st-stage	2nd-stage: 2-naphthol in mol/mol			
		0 (ctrl)	0.051	0.103	0.205
pH		3.35	3.34	3.18	3.26
Volume (ml)	2532	655	787.5	789.6	790
*Monomeric* (g/l)					
Glucose	0.38	3.04	2.45	2.70	2.50
Mannose	1.55	3.69	3.52	3.05	3.17
*Total* (g/l)					
Glucose	3.35	4.07	3.50	3.62	3.52
Mannose	14.47	3.30	3.45	2.65	2.91
Acetic acid	0.68	2.01	1.68	1.83	1.74
HMF	0.16	3.26	2.74	3.12	2.89
Furfural	0.24	0.45	0.33	0.28	0.28
2-Naphthol	0	0	0	0.044	0.062
*Monomeric* (g/kg raw biomass)					
Glucose	2.61	5.40	5.24	5.78	5.36
Mannose	10.67	6.55	7.52	6.53	6.80
* Total* (g/kg raw biomass)					
Glucose	22.98	7.23	7.49	7.76	7.54
Mannose	99.42	5.87	7.37	5.69	6.24
Acetic acid	4.64	3.58	3.60	3.92	3.72
HMF	1.10	5.8	5.85	6.70	6.20
Furfural	1.62	0.62	0.71	0.60	0.59
2-Naphthol	0	0	0	0.10	0.13

### Simultaneous saccharification and fermentation (SSF)

A previous study showed that 2-naphthol at concentrations higher than 0.1 g/l can inhibit the fermentation of glucose to ethanol [27]. In order to investigate whether the usage of 2-naphthol in the steam explosion pretreatment of spruce wood can also enhance the ethanol yield or whether it inhibits the fermentation, SSF experiments were performed. SSF experiments were conducted with extensively washed solids, filtered pretreated solids, and the whole second stage pretreatment slurry at final cellulose concentrations of 1 and 5 % w/w to determine the influence of inhibitors and 2-naphthol at different concentrations on the fermentation. Detoxification and neutralization of the pretreated solids before the subsequent bioprocessing can be very expensive [40] and avoidance of these additional processing steps would be beneficial.

#### SSF with 1% w/w cellulose

We first studied the simultaneous saccharification at a cellulose concentration of 1% w/w, which is similar to the conditions in the enzymatic hydrolysis experiments and quantified potential inhibitors in the fermentation broth.

To determine the amount of 2-naphthol present in the fermentation broth, the latter was extracted with chloroform. Furthermore, the concentrations of the potentially inhibiting compounds acetic acid, HMF, and furfural were quantified (Table 3). Only a small amount of 2-naphthol that was present during the fermentation derived from the pretreatment hydrolyzate (see Table 2). Most of the unreacted 2-naphthol was extracted from the biomass itself (see Table 4). The washing of the solids with water did hardly remove the remaining 2-naphthol, which can be explained by the very low water solubility of 2-naphthol (0.74 g/l at 25 °C [41]). Thus, the 2-naphthol concentrations were similar in all three fermentation modes, but were increasing with increasing 2-naphthol loadings in the pretreatment. In contrast, the concentrations of acetic acid, HMF, and furfural were virtually independent of the 2-naphthol loading in the pretreatment (see Table 2) but increased with the amount of hydrolyzate introduced to the fermentation. The concentrations of these inhibitors were about twice as high in the whole slurry fermentations compared to the fermentations using filtered solids.

**Table 3:** Final concentrations of inhibitory substances in SSF experiments with 1% w/w cellulose

2-Naphthol (mol/mol C_9_)	Concentration (g/l)			
	Acetic acid	HMF	Furfural	2-Naphthol
*Washed solids*				
0 (control)	0	0	0	0
0.051	0	0	0	0.013
0.103	0	0	0	0.026
0.205	0	0	0	0.067
*Filtered solids*				
0 (control)	0.123	0.200	0.021	0
0.051	0.107	0.175	0.021	0.017
0.103	0.110	0.188	0.017	0.027
0.205	0.108	0.179	0.017	0.072
*Whole slurry*				
0 (control)	0.244	0.395	0.042	0
0.051	0.233	0.380	0.046	0.017
0.103	0.244	0.416	0.037	0.030
0.205	0.236	0.393	0.037	0.076

2-Naphthol was quantified after extraction of the whole fermentation slurry with chloroform

**Table 4:** Residual 2-naphthol in biomass

	2nd-stage: 2-naphthol in mol/mol			
	0 (ctrl)	0.051	0.103	0.205
*Residual 2-naphthol* (mg/g pretreated biomass)				
Washed solids	0	0.173	0.294	0.780
Filtered solids	0	0.203	0.333	0.858

**Fig. 4:**
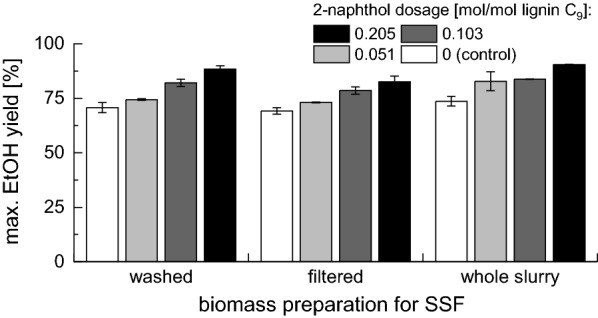
Influence of 2-naphthol dosage on maximal ethanol yield in SSF with 1% w/w cellulose. Ethanol yield expressed as % of theoretical yield

**Fig. 5:**
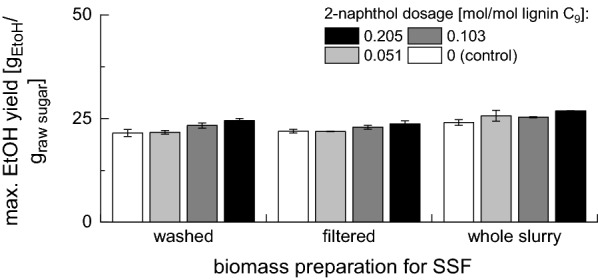
Influence of 2-naphthol dosage on maximal ethanol yield in SSF with 1% w/w cellulose

The final ethanol yields achieved in SSF experiments with 1% w/w final cellulose concentration are presented in Fig. 4 [the yields based on the raw biomass (non-pretreated) are presented in Fig. 5]. For all three fermentation modes, the ethanol yield was increasing with increasing 2-naphthol dosage. The ethanol yields in the controls pretreated without 2-naphthol were $70.7\pm 2.3$%, $69.2\pm 1.5$% and $73.6\pm 2.2$% ($21.5\pm 0.8$%, $21.9\pm 0.5$% and $24.1\pm 0.7$% based on the raw biomass) for the washed, filtered and whole slurry biomass, respectively. At the highest 2-naphthol dosage, the yields increased to $88.4\pm 1.7$, $82.5\pm 2.8$% and $90.4\pm 0.3$% ($24.5\pm 0.5$%, $23.7\pm 0.7$% and $26.8\pm 0.0$% based on the raw biomass), respectively. This shows that the addition of 2-naphthol to the pretreatment of spruce wood not only enhances the enzymatic digestibility but also the ethanol yields during SSF as no inhibition of the fermentation by residual 2-naphthol or other inhibitors occurred.

#### SSF with 1% w/w cellulose and hydrolysate from first stage

To valorize all carbohydrates from the biomass, SSF experiments with a cellulose loading of 1% w/w were performed with the addition of the first stage hydrolysis liquor. The concentration of the potentially inhibitory substances acetic acid and furfural were approximately twice as high compared to the experiments without the hydrolysate, whereas the concentrations of HMF were similar (see Table 5). The concentration of residual 2-naphthol was identical to the experiments without the hydrolysis liquor from the first stage, since 2-naphthol was only added to the second pretreatment stage.

**Table 5:** Final concentrations of potential inhibitory substances in SSF experiments with 1% w/w cellulose concentration with added hydrolysate from the first pretreatment stage

2-Naphthol (mol/mol C_9_)	Concentration (g/l)			
	Acetic acid	HMF	Furfural	2-Naphthol
*Washed solids*				
0 (control)	0.157	0.037	0.055	0
0.051	0.167	0.040	0.058	0.013
0.103	0.171	0.040	0.060	0.026
0.205	0.165	0.039	0.057	0.067
*Filtered solids*				
0 (control)	0.279	0.237	0.076	0
0.051	0.269	0.213	0.078	0.017
0.103	0.272	0.226	0.073	0.027
0.205	0.268	0.217	0.073	0.072
*Whole slurry*				
0 (control)	0.399	0.432	0.096	0
0.051	0.395	0.418	0.102	0.017
0.103	0.406	0.455	0.094	0.030
0.205	0.396	0.431	0.093	0.076

2-Naphthol was quantified after extraction of the whole fermentation slurry with chloroform

**Fig. 6:**
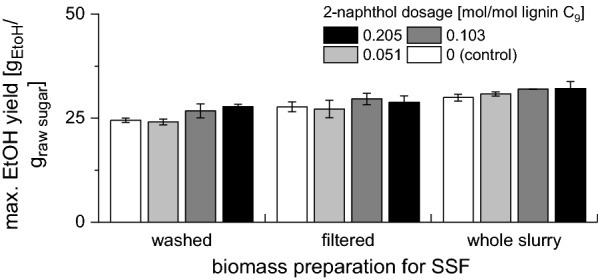
Influence of 2-naphthol dosage on maximal ethanol yield in SSF with 1% w/w cellulose and added hydrolysate from the first pretreatment stage

The final ethanol yields based on the raw biomass (non-pretreated) achieved with 1% w/w final cellulose concentration and added first stage hydrolysate are presented in Fig. 6. For all three fermentation modes, the ethanol yield increases with increasing 2-naphthol dosage. The ethanol yields in the controls pretreated without 2-naphthol were $24.5\pm 0.5$%, $27.7\pm 1.2$% and $30.0\pm 0.8$% for the washed, filtered and whole slurry biomass, respectively. At the highest 2-naphthol dosage, the yields increased to $27.8\pm 0.6$%, $28.8\pm 1.6$% and $32.1\pm 1.7$% for the washed, filtered and whole slurry biomass, respectively).

This shows that the added hydrolysate from the first pretreatment stage does not lead to inhibition of the fermentation, but increases the ethanol titers in SSF due the additional soluble sugars.

#### SSF with 5% w/w cellulose

To investigate the influence of higher solid loadings, SSF experiments with a cellulose concentration of 5% w/w were performed, which equals $\sim$ 10% solid loading. Due to the dilution of the biomass slurry in the steam gun resulting from steam condensation during the steam pretreatment, higher concentrations were not possible. Sugar yields in enzymatic hydrolysis and ethanol yields in SSF generally decrease with higher solid loadings due to product inhibition, mass transfer issues, and other reasons, which is also termed the solid effect [42, 43]. Furthermore, the concentrations of fermentation inhibitors are increasing with higher solid loadings, making high solid fermentations challenging.

**Fig. 7:**
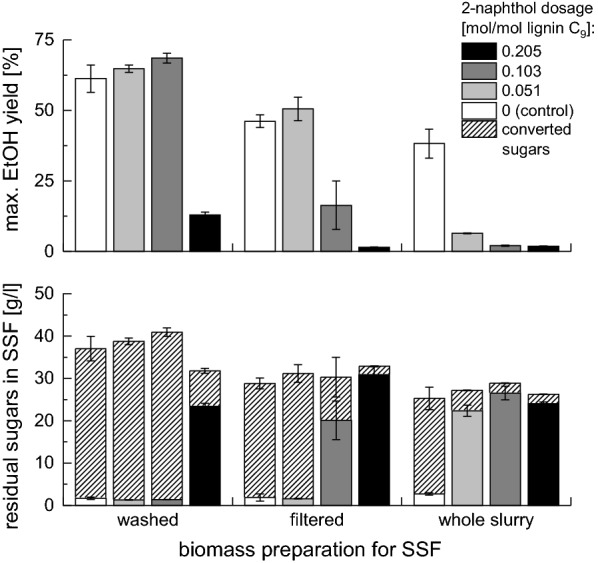
Influence of 2-naphthol dosage on maximal ethanol yield in SSF with 5% w/w cellulose and residual sugar concentrations in SSF liquors. EtOH yield expressed as % of theoretical yield. Residual sugar concentration = sum of residual monomeric sugars in supernatant. This includes introduced sugars, which were < 0.5 g/l in the whole slurry and < 0.25 g/l in the filtered biomass. Sugars, which were converted to EtOH are shown streamlined

Figure 7 shows the ethanol yields in the high solid SSF experiments as well as the residual monomeric sugar concentrations in the fermentation broth (the ethanol yields based on a beginning carbohydrate content as well as the final ethanol concentrations are presented in Additional files 3 and 4). For an estimation of the enzymatic hydrolysis yield, the measured ethanol concentrations were converted to the corresponding sugar concentrations assuming stoichiometric conversion to ethanol. The concentrations of potentially inhibitory substances in the fermentation broth are presented in Table 6.

**Table 6:** Final concentrations of potential inhibitory substances in SSF experiments with 5% w/w cellulose

2-Naphthol (mol/mol C_9_)	Concentration (g/l)			
	Acetic acid	HMF	Furfural	2-Naphthol
*Washed solids*				
0 (control)	0	0	0	0
0.051	0	0	0	0.067
0.103	0	0	0	0.128
0.205	0	0	0	0.334
*Filtered solids*				
0 (control)	0.617	1.000	0.107	0
0.051	0.537	0.875	0.105	0.086
0.103	0.551	0.940	0.084	0.135
0.205	0.538	0.896	0.085	0.358
*Whole slurry*				
0 (control)	1.218	1.975	0.211	0
0.051	1.166	1.898	0.229	0.086
0.103	1.220	2.081	0.187	0.152
0.205	1.180	1.965	0.187	0.381

2-Naphthol was quantified after extraction of the whole fermentation slurry with chloroform

Compared to the SSF results with 1% w/w cellulose, the ethanol yields were generally lower for all samples and did not exceed 70%. In the samples which were not inhibited by 2-naphthol, the ethanol yields were around 10–15% lower than in the SSF experiments with 1% w/w cellulose thereby showing the expected solid effect [43, 44]. The highest ethanol yields could be achieved for SSF of washed solids. The yields increased with increasing 2-naphthol dosage up to a 2-naphthol loading of 0.103 mol /mol lignin C_9_-unit in the pretreatment from 61.3 ± 4.8% for the control to 68.5 ± 1.7%. For the sample pretreated with 0.205 mol 2-naphthol/mol lignin C_9_-unit, a much lower ethanol yield was achieved (12.9 ± 1.1%). Correspondingly, a high residual sugar concentration was measured in this sample, which contained 0.334 g/L naphthol. Thus, this 2-naphthol concentration strongly inhibits the fermentation, but to a much lesser extent the enzymatic hydrolysis, whereas a 2-naphthol concentration of 0.128 g/l is tolerable for the fermenting yeast. It is reported that in pure glucose fermentations a dosage of 0.4 g/l 2-naphthol completely inhibits the growth of the microorganisms while with a dosage of 0.2 g/l 2-naphthol the ethanol yield is reduced to 40% [27]. The yield of 13% we determined in the presence of 0.334 g/l 2-naphthol is in good agreement with this study. For the biomass pretreated with the highest amount of 2-naphthol, also the sum of the residual sugars and the sugars converted to ethanol was lower than in the other samples thereby indicating that the residual 2-naphthol did also negatively influence the enzymatic hydrolysis.

In SSF of filtered but unwashed solids, generally lower yields were achieved than in the washed solids conversions. The highest ethanol yield of $50.5\pm 4.2$% was determined for the sample pretreated with the lowest 2-naphthol dosage of 0.051 mol/mol lignin C$_9$ which was slightly higher than the yield of the control ($46.2\pm 2.2$%). Very low residual sugar concentrations in these two samples show that the ethanol yield was limited by the enzymatic cellulose conversion. At the highest 2-naphthol dosage in the pretreatment, almost no ethanol (< 2%) was produced in SSF; while with the second highest 2-naphthol dosage, a yield of 16.3 ± 8.6% ethanol was achieved. The residual 2-naphthol concentration of 0.135 g/l in the latter case is similar to the concentration in the sample with the washed solids pretreated under identical conditions. However, also acetic acid, HMF and furfural were introduced to the fermentation broth with the filtered solids. While both levels of these two types of inhibitors were well tolerated by the fermenting yeast when they were present individually, the combination led to a severe inhibition, showing a synergistic effect of the inhibitors. Interestingly, when the fermentation is prolonged from 10 to 18 days, the inhibitory effect could be overcome and similar ethanol yields as in the samples with lower 2-naphthol dosages were reached (see Additional file 5: Figure S5). The sum of residual sugars and the sugars converted to ethanol are generally similar but slightly higher for the samples with higher 2-naphthol dosage, which shows that 2-naphthol enhances the hydrolysis in all samples and the residual 2-naphthol does not inhibit the hydrolysis. However, the estimated sugar yields were higher in the samples with the washed solids, indicating that enzymatic hydrolysis is inhibited by HMF, furfural, and acetic acid as also known from literature [45].

The whole slurry experiments with a cellulose concentration of 5% showed for all different 2-naphthol concentrations very low ethanol yields (< 10%) and a yield of $38.2\pm 5.1$% for the control sample without 2-naphthol. Except for the control, very high residual sugar concentrations were measured in the supernatant. This shows that in all samples pretreated with 2-naphthol, the enzymes were active but the fermentation was inhibited. In the control sample, where only low residual sugar concentrations were measured, the ethanol production was limited by the enzymatic cellulose conversion. Again the synergistic effect of the inhibitors was evident: the concentrations of acetic acid, furfural, and HMF were the highest in the whole slurry experiments, thus a lower amount of 2-naphthol could be tolerated. The negative effect of the inhibitors is evident in the estimated enzymatic hydrolysis yields; in the whole slurry experiments, they are lower than in the other experiments.

## Conclusion

A two-stage pretreatment greatly enhances recovery of hemicellulosic sugars from spruce wood. However, the glucose yield in the enzymatic hydrolysis is reduced and the yield enhancing effect of 2-naphthol is less pronounced in comparison to a one-stage pretreatment. Adding a catalyst like sulfuric acid could further increase the yield in the enzymatic hydrolysis as well as the recovery of hemicellulosic sugars. In low solid SSF experiments, the ethanol yields are higher than the corresponding sugar yields in enzymatic hydrolysis. Whole slurry SSF with 1% w/w cellulose resulted in an ethanol yield of $\sim$ 90% for biomass pretreated with 0.205 mol 2-naphthol/mol lignin C$_9$-unit and $\sim$ 70% for the control sample pretreated without 2-naphthol. However, with higher solid loadings of 5% w/w cellulose, the ethanol yields were in general lower due to the solid effect and due to a synergistic inhibition by HMF, furfural, acetic acid and residual 2-naphthol. The higher the concentration of HMF, furfural, and acetic acid, the lower were the concentrations of residual 2-naphthol that were tolerated by the yeast.

## Additional files

**Additional file 1: Figure S1.** CAD drawing of the steam gun with the installed filtration device (blue) on top of the lower ball valve.

**Additional file 2: Figure S2.** Recovery of sugars from cellulose and hemicellulose in the first stage pretreatment hydrolysates.

**Additional file 3: Figure S3.** Influence of 2-naphthol dosage on maximal ethanol yield in SSF with 5% w/w cellulose

**Additional file 4: Figure S4.** Influence of 2-naphthol dosage on final ethanol concentration in SSF with 5% w/w cellulose.

**Additional file 5: Figure S5.** Influence of 2-naphthol dosage on EtOH yield in SSF with 5% w/w cellulose. Ethanol yield expressed as % of theoretical yield. Biomass preparations for SSF: whole slurry; vacuum filtered solids; washed solids. Pretreatment conditions: First stage: T = 180 °C, log *R *_0_= 3.75; Second stage: T = 230 °C, log *R *_0_ = 5, 0/0.051/0.103/0.205 mol 2-naphthol/mol lignin C_9_-unit. SSF conditions: T = 37 °C, 5% w/w cellulose, 60 FPU/g cellulose, OD_600_(t = 0) = 0.4.

## Abbreviations

AFEX: ammonia fiber expansion
FPU: filter paper unit
GC/MS: gas chromatography/mass spectrometry
HMF: 5-hydroxymethylfurfural
HWP: hot water pretreatment
SE: steam explosion pretreatment
SSF: simultaneous saccharification and fermentation
SPORL: sulfite pretreatment pretreatment to overcome recalcitrance
