# Supplementary figures and images for: Two-stage steam explosion pretreatment of softwood with 2-naphthol as carbocation scavenger
Source: Biotechnol Biofuels. 2019 Feb 21;12:37. doi: 10.1186/s13068-019-1373-3 (PMC6383249; doi:10.1186/s13068-019-1373-3)

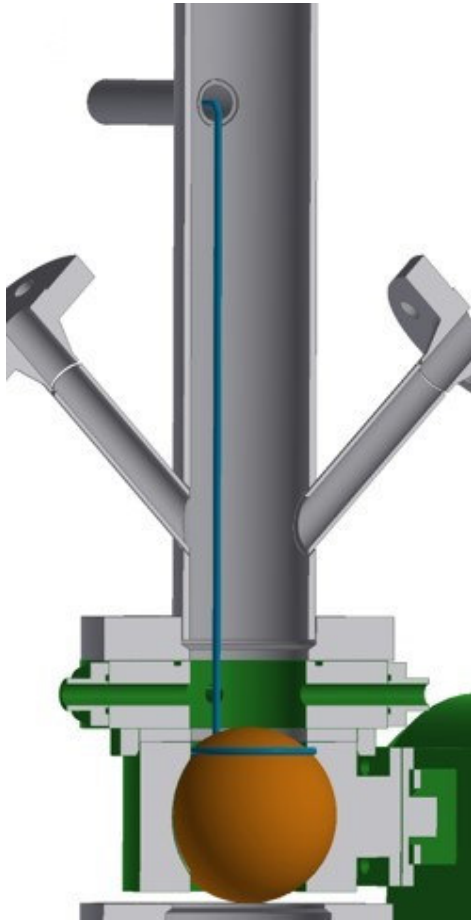

Supplement: Supplementary file 1 — Additional file 1: Figure S1. CAD drawing of the steam gun with the installed filtration device (blue) on top of the lower ball valve. [file 13068_2019_1373_MOESM1_ESM.pdf]

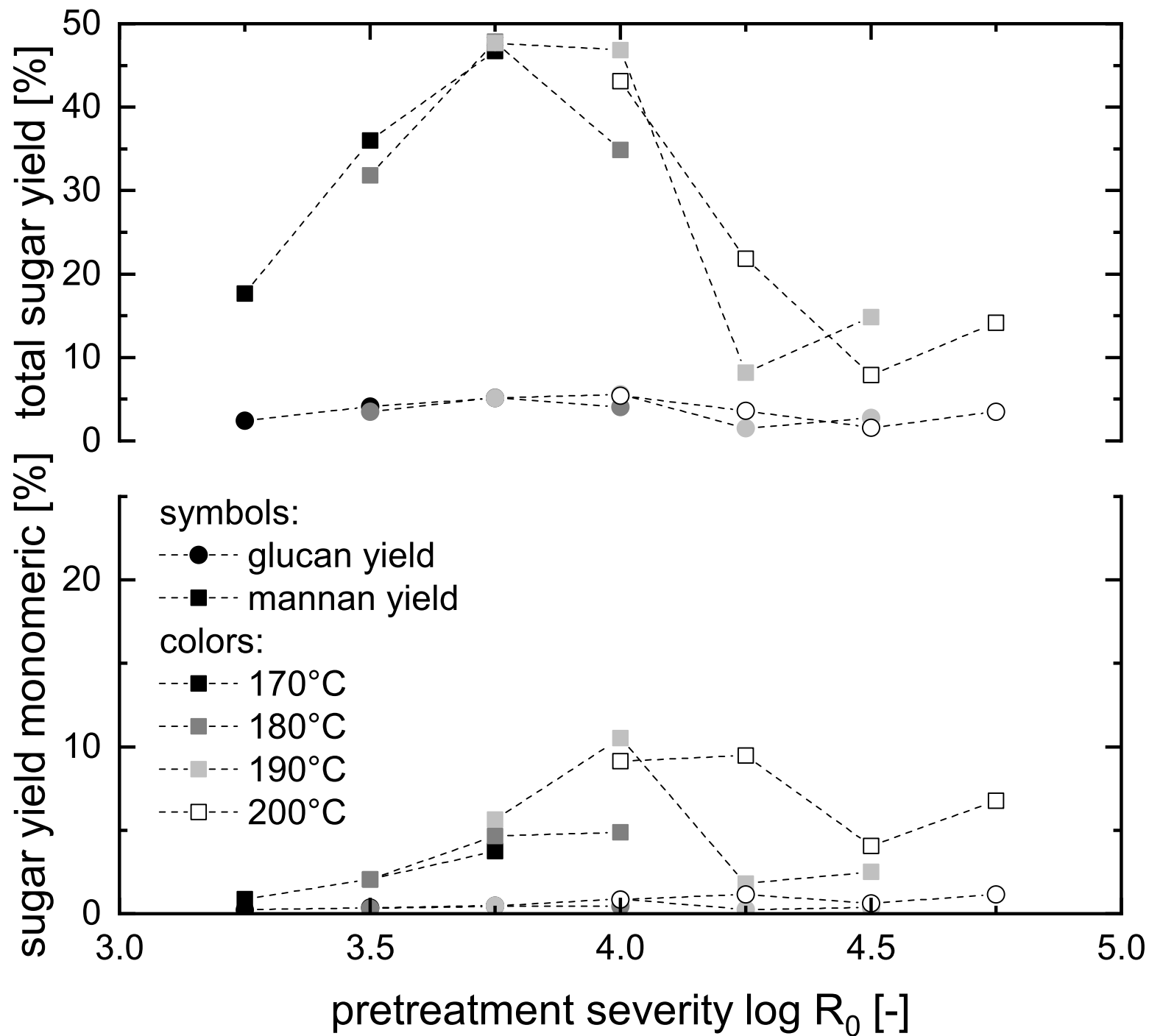

Supplement: Supplementary file 2 — Additional file 2: Figure S2. Recovery of sugars from cellulose and hemicellulose in the first stage pretreatment hydrolysates. [file 13068_2019_1373_MOESM2_ESM.pdf]

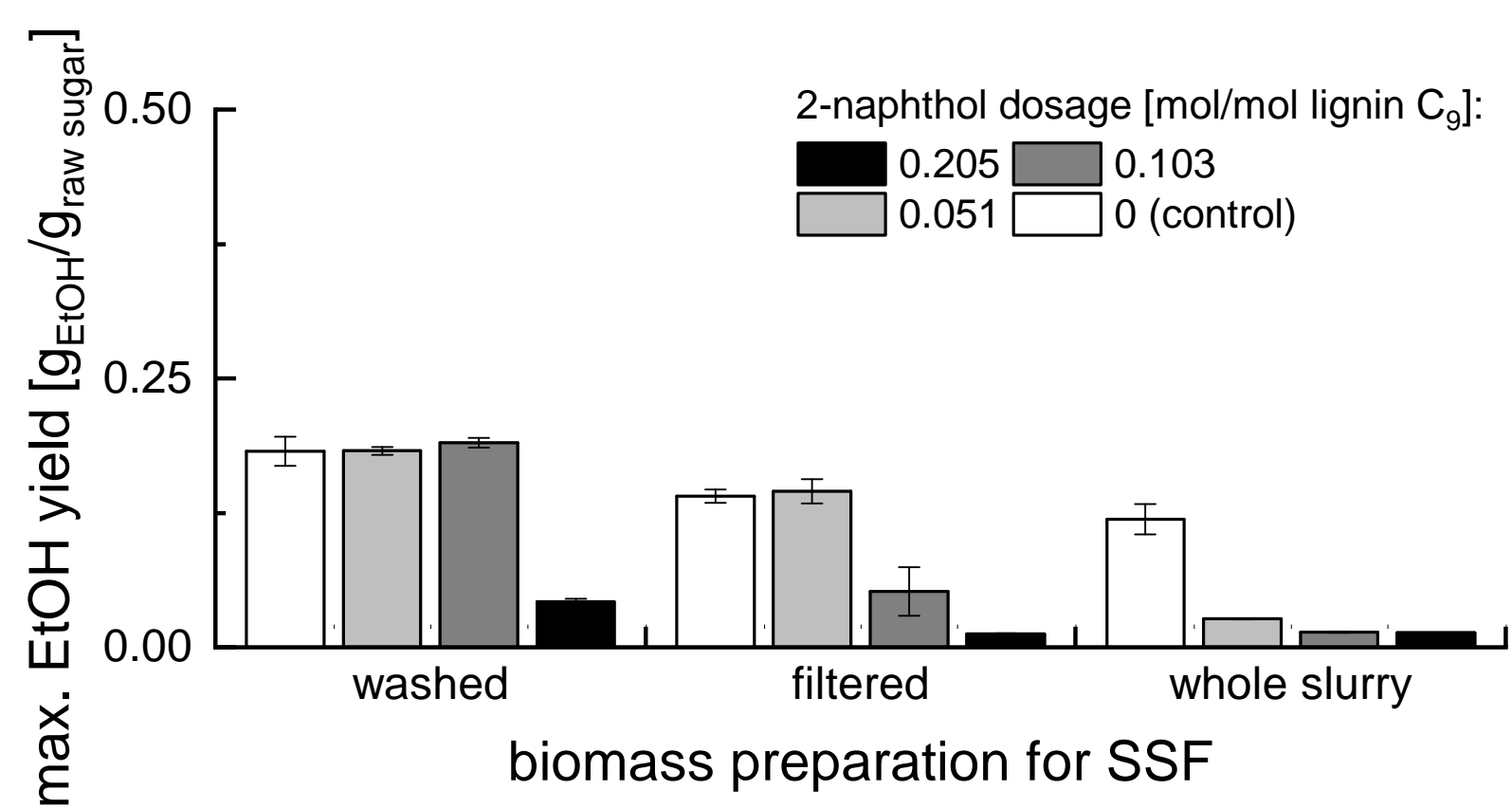

Supplement: Supplementary file 3 — Additional file 3: Figure S3. Influence of 2-naphthol dosage on maximal ethanol yield in SSF with 5% w/w cellulose [file 13068_2019_1373_MOESM3_ESM.pdf]

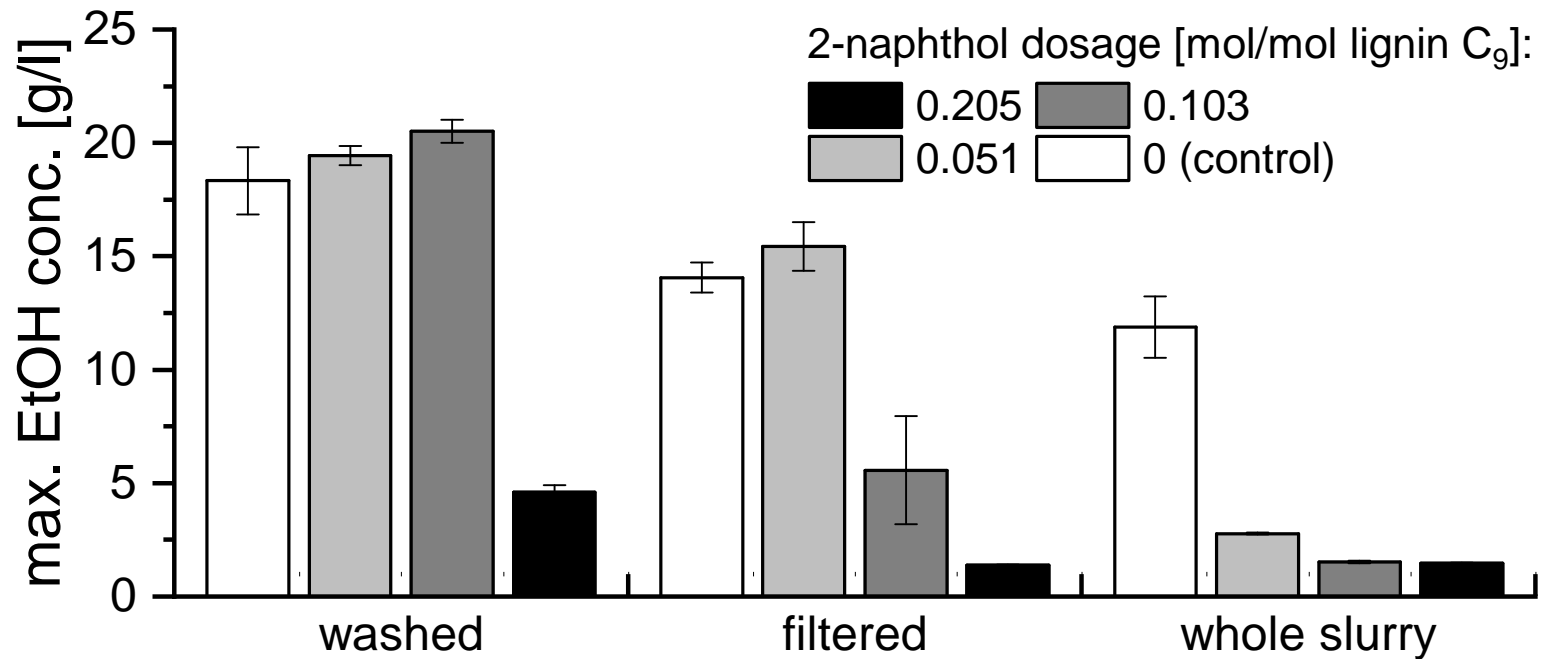

Supplement: Supplementary file 4 — Additional file 4: Figure S4. Influence of 2-naphthol dosage on final ethanol concentration in SSF with 5% w/w cellulose. [file 13068_2019_1373_MOESM4_ESM.pdf]

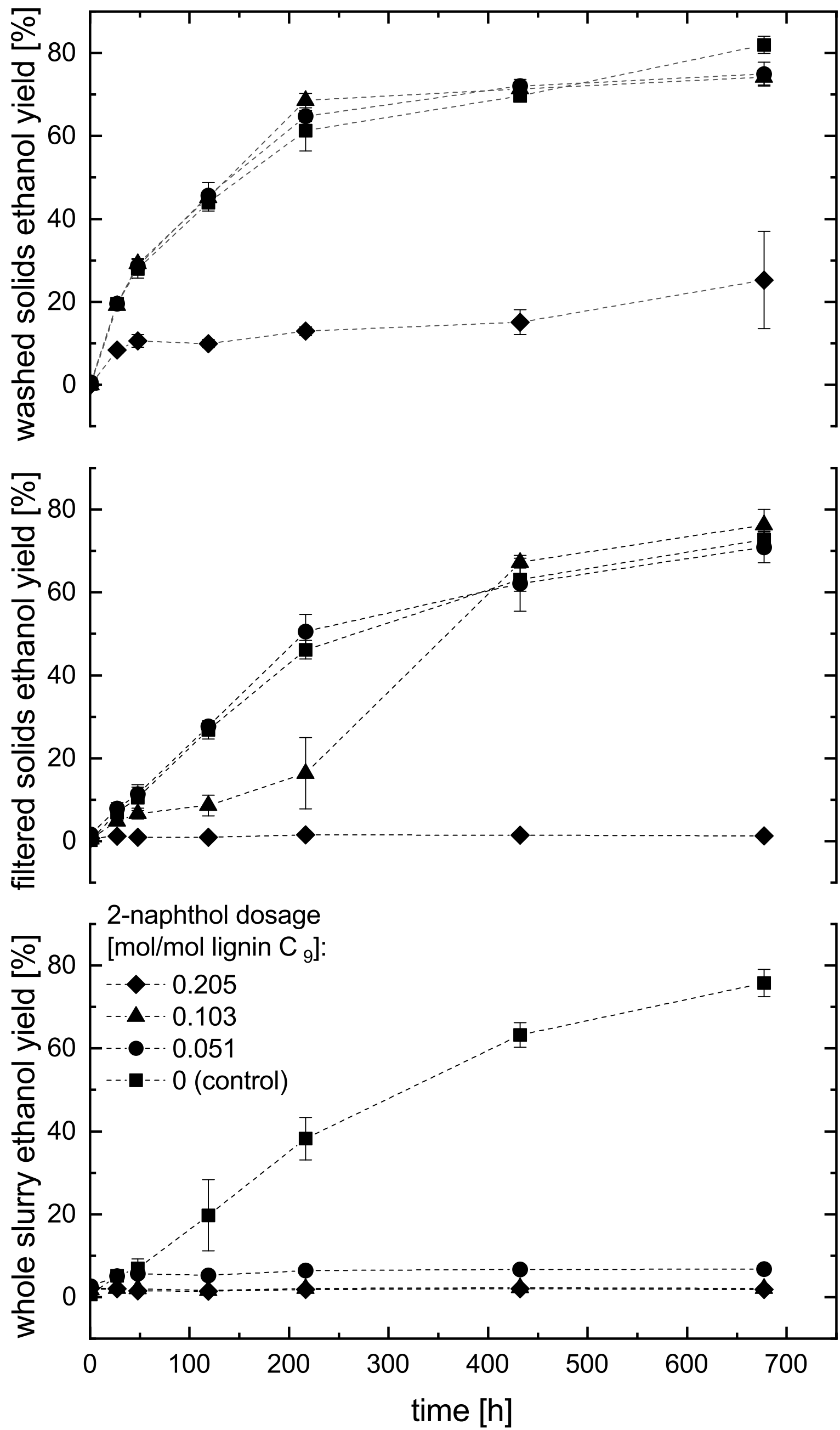

Supplement: Supplementary file 5 — Additional file 5: Figure S5. Influence of 2-naphthol dosage on EtOH yield in SSF with 5% w/w cellulose. Ethanol yield expressed as % of theoretical yield. Biomass preparations for SSF: whole slurry; vacuum filtered solids; washed solids. Pretreatment conditions: First stage: T = 180 °C, log R 0= 3.75; Second stage: T = 230 °C, log R 0 = 5, 0/0.051/0.103/0.205 mol 2-naphthol/mol lignin C9-unit. SSF conditions: T = 37 °C, 5% w/w cellulose, 60 FPU/g cellulose, OD600(t = 0) = 0.4. [file 13068_2019_1373_MOESM5_ESM.pdf]
